# Cellular Pathways in Response to Ionizing Radiation and Their Targetability for Tumor Radiosensitization

**DOI:** 10.3390/ijms17010102

**Published:** 2016-01-14

**Authors:** Patrick Maier, Linda Hartmann, Frederik Wenz, Carsten Herskind

**Affiliations:** Department of Radiation Oncology, Universitätsmedizin Mannheim, Medical Faculty Mannheim, Heidelberg University, Theodor-Kutzer-Ufer 1-3, Mannheim 68167, Germany; Linda.Hartmann@web.de (L.H.); frederik.wenz@umm.de (F.W.); carsten.herskind@medma.uni-heidelberg.de (C.H.)

**Keywords:** radiotherapy, radioresistance, radiosensitization, double strand break, DNA repair, apoptosis, mitotic catastrophe, senescence

## Abstract

During the last few decades, improvements in the planning and application of radiotherapy in combination with surgery and chemotherapy resulted in increased survival rates of tumor patients. However, the success of radiotherapy is impaired by two reasons: firstly, the radioresistance of tumor cells and, secondly, the radiation-induced damage of normal tissue cells located in the field of ionizing radiation. These limitations demand the development of drugs for either radiosensitization of tumor cells or radioprotection of normal tissue cells. In order to identify potential targets, a detailed understanding of the cellular pathways involved in radiation response is an absolute requirement. This review describes the most important pathways of radioresponse and several key target proteins for radiosensitization.

## 1. Introduction

Radiotherapy is an integral component of tumor treatment, providing a locoregional therapy that complements local and systemic treatment by surgery and chemotherapy, respectively. Thus, radiotherapy is applied in at least 50% of all cancer patients treated with curative intent [1], resulting in a cure rate of about 40% [2,3]. However, the effect of radiotherapy is limited by the radioresistance of the tumor cells to the applied doses in a fractionated regimen [4,5] and by adverse reactions in the normal tissues surrounding the tumor. The importance of the therapeutic window between tumor control and normal tissue damage was already pointed out in the early days of radiotherapy [6]. Although improved treatment modalities [7,8,9,10,11,12,13] and fractionated irradiation led to an increase in the efficiency of radiotherapy and to a decrease of normal tissue complications [14,15], further broadening of the therapeutic window is clinically important. Two strategies might be followed: (1) radiosensitization of the tumor cells without sensitizing normal tissue cells; (2) specific radioprotection of normal tissue cells. For both approaches, a detailed understanding of the cellular pathways involved in the cellular response to irradiation is mandatory in order to identify molecular targets for medical intervention. In this review, the basic properties of ionizing radiation will be introduced, and the various cellular pathways involved in the cellular radiation response leading to survival or induction of either cell death or senescence will be described. Furthermore, promising target proteins for radiosensitization will be highlighted. Current strategies for radioprotection of normal tissue have been reviewed recently [16].

## 2. Properties and Biological Effects of Ionizing Radiation

Different qualities of ionizing radiation are characterized by the ionization density of their tracks quantified by the mean energy deposited per track length, termed linear energy transfer (LET) [15,16]. X-rays, γ-rays and electrons with typical LET values in the range of 0.2 to 5 keV/µm are called low-LET radiation, while neutrons and heavier ions with typical LET values in the range of 50 to 200 keV are called high-LET radiation and have increased relative biologic effectiveness (RBE) [17].

The main cellular target of ionizing radiation is the DNA. The track of a charged particle (an electron or an ion) may pass through and ionize DNA directly (direct action) or ionize water molecules in the vicinity, thereby producing highly reactive OH^•^ radicals, which can diffuse to DNA and react with the target molecule (indirect action). Other aqueous free radicals and reactive oxygen species (ROS), including O_2_^•−^ and H_2_O_2_, are less important than OH^•^ for producing lethal DNA damage [18,19]. Direct action dominates for high-LET radiations, where only three to four dense tracks of ionizations cross each nucleus per Gy (Gray) of energy deposited. By contrast, the ratio between indirect and direct action is approximately 3:1 for low-LET radiation, which deposits approximately one thousand electron tracks in the nucleus per unit dose of 1 Gy. Chemical reactions in DNA induced either by direct ionization or indirectly by OH^•^ radicals may damage a base, interrupt the sugar-phosphate backbone, resulting in a single- or in a double-strand break (SSB or DSB) or produce a DNA crosslink. Approximately 3000 damaged bases, 1000 SSBs and 40 DSBs are induced in a cell by an X-ray dose of 1 Gy [20]. Nevertheless, base damage and SSB are of minor relevance for cell survival, since these lesions are essentially all repaired by the highly efficient base excision repair (BER) mechanism [21]. Even the vast majority of DSBs induced by low-LET radiation are also repaired (see Section 3.2). However, a small fraction (<5%) of DSBs induced by low-LET radiation cannot be repaired because of their higher complexity and constitute the most severe DNA damage after irradiation leading to cell death, senescence, mutations or genomic instability. DSBs produced by high-LET radiations are mostly of the complex type, and little DSB repair occurs.

## 3. Signaling Pathways of the Cellular Radiation Response

### 3.1. p53 as a Guardian of Genomic Stability: Repair or Cell Death

The first step in the DNA damage response (DDR) is sensing of the DNA damage by ATM (ataxia-telangiectasia mutated) and ATR (ataxia-telangiectasia and RAD3-related), which are the initiating kinases phosphorylating and activating various downstream proteins [22,23]. ATM is the master regulator of DDR sensing DSBs induced by ionizing irradiation [24]; ATR is mostly active after SSBs or stalling of replication forks caused by UV light or hydroxyurea [25].

**Figure 1:**
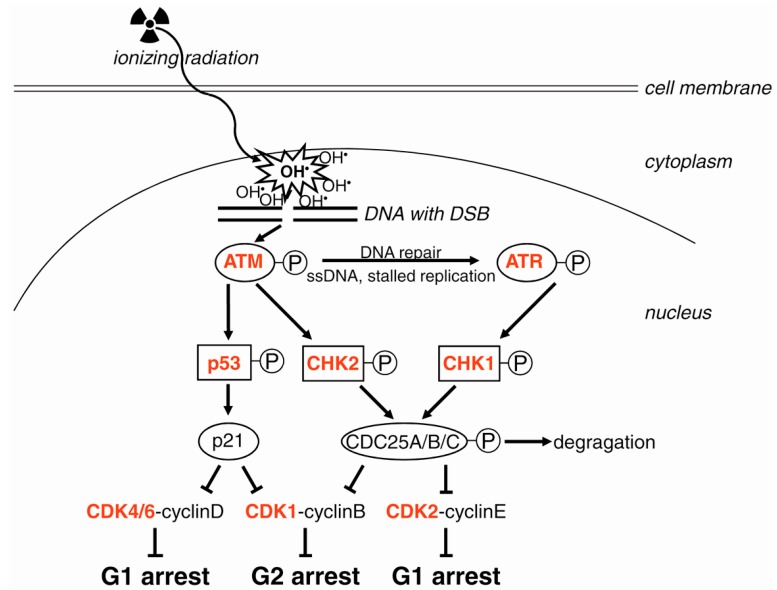
Induction of cell cycle arrest after irradiation. The hydroxyl radical is the most important aqueous radical induced by ionizing radiation (symbolized by the sinuous arrow and the trefoil) affecting the integrity of DNA (parallel lines) by induction of double strand breaks (DSB, gap in DNA). Subsequently, the ATM (ataxia-telangiectasia mutated) kinase is activated by phosphorylation (encircled P) and, in turn, phosphorylates p53. ATR (ataxia-telangiectasia and RAD3-related) is activated by single-stranded DNA and stalled replication forks arising from the repair process. Activated p53 acts as a transcription factor and causes the expression of the cyclin-dependent kinase (CDK) inhibitor p21, which induces cell cycle arrest during the G1 and G2 phases. On the other hand, activation of CHK1 and CHK2 (checkpoint kinase-1 and -2) leads to phosphorylation of the three CDC25 (cell division cycle 25) isoforms, resulting in its degradation. As a consequence, CDC25 no longer activates CDK2 or CDK1 (cyclin-dependent kinase), and thus, the cell cycle is stopped in the G1 or G2 phase, respectively. Arrows symbolize activation; bar-headed lines symbolize inhibition. Targets for radiosensitization are in red. See text for details.

Arrest of the cell cycle is an important part of DDR, facilitating DNA repair and maintenance of genomic stability [26]. Regulators of cell cycle arrest are activated by phosphorylation by ATM and ATR (Figure 1). The transducers CHK1 and CHK2 (checkpoint kinase-1 and -2) lie directly downstream of ATM [27]. The tumor suppressor protein p53 is a central stress protein in the DDR. ATM, CHK1 and CHK2 phosphorylate p53 at different positions [28], resulting in stabilization of p53 by dissociation from MDM2 (mouse double minute 2 homolog) and accumulation in the nucleus, where it acts as a transcription factor [29]. Under normal conditions, p53 is constitutively expressed, but is bound by the E3 ubiquitin ligase MDM2 and undergoes proteasomal degradation. The degree of DNA damage and the extent of modifications of p53 (e.g., phosphorylation, acetylation, methylation) determine which of the two alternative pathways are activated by p53, either cell survival or cell death [30]. Stronger DNA damage causes enhanced and prolonged activation of p53. A recent model predicts a “digital” response of p53 to DNA damage: the “arrester” p53 is phosphorylated at Ser 15; the “killer” p53 is additionally phosphorylated at Ser 46 and mediates apoptosis [31]. Moreover, the decision between cell cycle arrest and apoptosis is determined by the varying spectrum of p53 responsive genes in different cell lineages [32]. Due to this pivotal role in the decision of cell survival or cell death and genomic stability, p53 has been termed “the guardian of the genome” [33].

For cell cycle arrest, p53 induces transcription of p21 (CDKN1A, cyclin-dependent kinase inhibitor 1A) (Figure 1), and activated p53 phosphorylates and activates the p21 protein, resulting in inhibition of the activity of CDK4 and CDK6 and, thus, in G1 arrest [34]. Enhanced expression of p21 in response to activated p53 also blocks transition from the G2 to M phase; p21 binds the CDK1-cyclinB complex, thereby preventing its activation [35]. Additional faster cell cycle control is performed by CHK1 and CHK2, which phosphorylate the three isoforms of the CDC25 (cell division cycle 25) phosphatase, initiating their ubiquitination and degradation. The consequence is the inhibition of dephosphorylation and activation of CDK2-cyclinE and of CDK1-cyclinB, resulting in cell cycle arrest in the G1 phase or in the G2 phase, respectively [36].

### 3.2. Signaling for Double-Strand Break Repair

After cell-cycle arrest, DSBs are repaired mainly by two pathways, non-homologous end joining (NHEJ) and homologous recombination (HR) [37]. NHEJ can repair DSBs throughout the cell cycle, but is mainly used during the G1 phase. NHEJ accurately repairs simple DSBs with complementary overhangs and phosphorylated and hydroxylated 5′ and 3′ ends, respectively. However, NHEJ will become inaccurate if DNA ends require processing before ligation; then, NHEJ causes short additions or deletions of DNA sequences and, thus, possibly a loss of genetic information. Furthermore, misrepair due to ligation of DNA ends of different DSBs will cause translocations, rearrangements and di- or acentric chromosomes, which result in the formation of micronuclei and, subsequently, in aneuploidy [38]. In contrast to the error-prone NHEJ, HR is error free, but requires an undamaged sister chromatid for the recombination and is therefore only available in the late S and G2 phases.

The decision of which of the two pathways becomes activated is determined by the competition between protection and resection of the DNA ends. The Ku70–Ku80 heterodimer (Ku) senses the DSB, binds to the DNA ends, blocks 5′ end resection and holds both ends in close proximity, thus enabling direct rejoining of DNA ends by NHEJ [38]. Ku also activates 53BP1 (p53-binding protein 1) for additional protection of DSB ends against resection. Phosphorylation of H2AX on Ser 139 (γ-H2AX) in the nucleosome next to the breakpoint by ATM further stabilizes the DNA ends and serves as the scaffold for assembly of the DSB repair machinery, including PRKDC (protein kinase, DNA-activated, catalytic subunit) and Artemis [39]. Repair of the DSB is completed by ligation of both DNA ends by LIG4 (DNA ligase 4), XRCC4 (X-ray repair, complementing defective, in Chinese hamster, 4) and XLF (XRCC4-like factor) [37]. Recently, a function of the androgen receptor (AR) in the radiation response of prostate tumor cells was elucidated [40]. Dimerization of AR is induced by ionizing radiation; subsequently, it induced the expression of especially PRKDC and Ku70 required for NHEJ. PRKDC further stimulates AR, thus establishing a positive feedback [41,42]. However, not only activity, but also expression of AR is upregulated after radiation, resulting in increased radioresistance of prostate cancer cells during radiotherapy [43].

Repair by HR is initiated by the sensing MRN complex consisting of MRE11, RAD50 and NBS1 [44]. Similar to NHEJ, the signal is transmitted to the neighboring ATM and ATR, which phosphorylate various downstream actors. For DNA end processing, resection of double-stranded ends is initiated by the MRN complex (nicking activity of MRE11 at a distance from dsDNA ends) in combination with CTIP (CTBP (C-terminal binding protein)-interacting protein) [37]. Due to the strand degradation in the 3′ to 5′ direction, Ku is released from DSB ends. Then, HR can be initiated by annealing of the generated single-stranded DNA to the unwound sister chromatid. A pivotal role in this process is played by RAD51, which forms nucleoprotein filaments on ssDNA as a prerequisite for strand exchange. RAD51 forms a complex BRCA2 (breast cancer 2), which becomes activated by phosphorylation after DSB, enabling the binding of RAD51 to ssDNA [45].

HR is restricted to the S and G2 phases by phosphorylation of CTIP (Ser 327 and Thr847) by specific CDKs, enabling CTIP to complex with MRN and BRCA1 (breast cancer 1 gene) and to activate DSB resection [37]. Furthermore, expression of CTIP is cell cycle dependent with low expression during the G1 phase, increased expression in the S/G2 phases and proteolytic degradation in the subsequent G1 phase [46]. A pivotal role in the choice of NHEJ or HR is played by BRCA1; it antagonizes 53BP1 at DSB ends during the S and G2 phases, thus impeding NHEJ and facilitating HR [47,48]. The interplay between the mediators 53BP1 and BRCA1 and the upstream sensing molecules Ku and the MRN complex is significant for the decision of whether NHEJ or HR is used for DNA repair [37].

If the DNA damage can be repaired completely, the cell will continue its cell cycle. In contrast, the consequence of improper DNA repair after irradiation is the onset of cell death, either by apoptosis, mitotic catastrophe or senescence. How the cell will die might be influenced by several parameters: primarily the cell type, the supply with oxygen, the cell cycle phase in which irradiation occurs and, very importantly, the dose and radiation quality [49]. Hematopoietic and lymphoid cells, and also leukemia cells, are particularly prone to rapid radiation-induced cell death by the apoptotic pathway. In most solid tumors, mitotic cell death (mitotic catastrophe) is as least as important as apoptosis, and in some cases, it is the only mode of cell death. In contrast, senescence is the fate of irradiated cells in the majority of normal tissues.

### 3.3. Apoptosis

**Figure 2:**
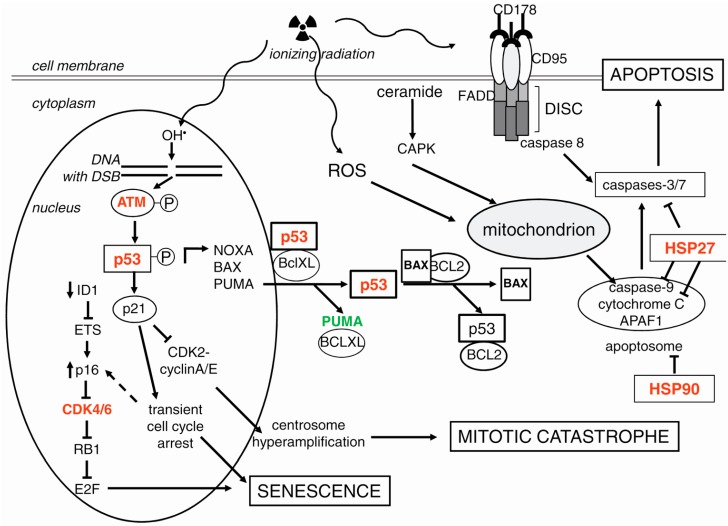
Cell death pathways after irradiation. Apoptosis is mostly induced by irradiation (symbolized by the sinuous arrows and the trefoil) in hematopoietic cells, or in cells of the mucosa in the gastro-intestinal tract, or in p53 wildtype tumor cells. DSB-dependent activation of p53 results in increased expression pro-apoptotic genes, inducing the intrinsic apoptotic pathway (see the text for details). Additionally, apoptosis might be induced by irradiation due to reactive oxygen species (ROS) production, or by activation of the second messenger ceramide, or the extrinsic apoptotic pathway. In contrast, the cell fate in most normal tissues is senescence induced by the p53/p21 and the p16/RB1 pathways, which result in cell-cycle arrest in the G1 phase and subsequent senescence (see the text for details). In p53-deficient cells, the blockage of CDK2-cyclinA/E by p21 is not functional; thus, centrosome hyper-amplification might occur, which is the prerequisite for mitotic catastrophe. This kind of cell death is caused by irradiation in most solid tumor entities. Arrows symbolize activation, the arrow with dotted line indicates a postulated effect; bar-headed lines symbolize inhibition. Targets for radiosensitization are in red, for radioprotection in green. See text for details.

Radiation induces mostly the intrinsic apoptotic pathway (mitochondrial release of cytochrome c and subsequent apoptosome formation), but depending on dose and cell type, the extrinsic apoptotic pathway (death receptor-mediated caspase activation) or the membrane stress pathway (ceramide production and subsequent second messenger signaling) might be the consequence of irradiation [50] (Figure 2).

The intrinsic apoptotic pathway is initiated by signaling following SSBs and DSBs if DNA repair is not successful [32]. The stronger and longer the activation of p53 as a key determinant in DDR, the higher the chances for apoptosis instead of growth arrest [51]. p53 can contribute to both the intrinsic mitochondria-mediated and the extrinsic death-receptor-mediated apoptosis.

#### 3.3.1. The Intrinsic Apoptotic Pathway

The control and regulation of apoptotic mitochondrial events occurs through members of the BCL2 (B-cell lymphoma 2) family of proteins [52], which govern mitochondrial membrane permeability and can be either pro-apoptotic or anti-apoptotic. Nuclear accumulation of p53 activates the expression of the pro-apoptotic BCL2 genes *PUMA* (p53-upregulated modulator of apoptosis), *BAX* (BCL2-associated X protein) and *NOXA* [53,54,55]. After its translocation to the cytoplasm, PUMA disrupts a complex by p53 and the anti-apoptotic protein BCL2L1 (BCL2-like 1 or BCL-XL). Liberated p53 dissolves the complex of the anti-apoptotic BCL2 and the pro-apoptotic BAX. Released BAX then triggers cell death by permeabilization of the outer mitochondrial membrane and subsequent release of cytochrome c [53,56]. Furthermore, ionizing radiation can directly enhance the production of O_2_^•−^ by mitochondria triggering the release of cytochrome c [57]. O_2_^•−^, but also other ROS, like H_2_O_2_ or OH^•^ radicals, can cause the release of Ca^2+^ from mitochondria [58], provoking various possible pro-apoptotic consequences: (1) loss of the mitochondrial membrane potential [59,60]; (2) release of proapoptotic mitochondrial proteins, which is coupled to stress response, known as the inner mitochondrial membrane (IMM) permeability transition [61]; (3) production of ROS due to binding of Ca^2+^ to cardiolipin in the IMM results in the oxidation of membrane phospholipids and proteins and, thus, in increased membrane permeability [62]; (4) dephosphorylation of pro-apoptotic BAD (BCL2-associated agonist of cell death) by the Ca^2+^/Calmodulin-dependent protein phosphatase calcineurin causing translocation of BAD from the cytoplasm to the mitochondria followed by release of cytochrome c from mitochondria [61,63].

The release of cytochrome c into the cytosol leads to the formation of the cytochrome c/APAF1 (apoptotic protease activating factor 1)/caspase-9 containing apoptosome complex [64]. The initiator caspase-9 then activates the effector caspases-3 and -7, thus inducing the post-mitochondrial-mediated caspase cascade [65]. The heat shock proteins (HSP) 27, 70 and 90 interfere with formation of the apoptosome; either by HSP27-mediated sequestering of cytochrome c [66] or by binding of HSP70 or HSP90 to APAF1 [67,68], and, therefore, inhibit the activation of procaspase-9. Thus, targeting one of these three HSPs in cancer cells is a promising approach for radiosensitization (Table 1).

#### 3.3.2. The Extrinsic Apoptotic Pathway

Radiation-induced apoptosis is also executed through the canonical extrinsic apoptotic pathway by signaling through death receptors (DRs), which belong to the tumor necrosis factor receptor (TNFR) super family [123,124]. Activation of p53 by radiation causes downstream transactivation of the receptor CD95, DR5 and the CD95 ligand (CD95L or CD178) [125,126]. Binding of CD178 to CD95 results in trimerization of CD95 and clustering of its intracellular death domain (DD). The DD recruits the adaptor protein FADD (FAS (FAS cell surface death receptor)-associated death domain) [126]. Subsequently, procaspase-8 interacts with the death effector domain (DED) of FADD, forming the death-inducing signaling complex (DISC). Activation of the initiator caspase-8 results again in activation of procaspase-3 and procaspase-7. In addition, downstream of CD95, activation of caspases can also proceed through the intrinsic mitochondria-dependent mechanisms [127].

**Table 1:** Targets of radiosensitizing approaches and the effected pathways. Only those references are stated describing the combination with irradiation.

Target	Substance	Radiosensitization of Cell Line/Tumor Entity	Comments	Reference
ATM	CP466722	HeLa (cervix carcinoma)	only in vitro results	[69]
ATM	KU-55933	various tumor cell lines HeLa, MCF-7, ovary cancer cells, bladder cancer cell, *etc.*	up to now no clinical trial	[70,71,72,73]
ATM	KU-60019	glioblastoma and glioblastoma-initiating cells	successor of KU-55933	[74,75,76]
			increased radiosensitivity in p53-deficient cells	[75,77]
ATR	NU6027	MCF-7 (breast carcinoma)	increased effects in combination with various chemotherapeutic drugs	[78]
BCR-ABL	imatinib	RT112 (transitional bladder cell carcinoma), H1299 (lung carcinoma), PANC1 (pancreatic adenocarcinoma), PC3 (prostate adenocarcinoma)	no increased radiation gut toxicity in an animal model with xenotransplantation of PC3	[79]
CDK1, 2, 4	flavopiridol (alvocidib)	various cancer cell lines and xenografts	successful clinical studies in combination with standard chemotherapeutic regimens	[80,81,82]
CDK1, 2, 9	AZD5438	A549, H1299, and H460 (non-small cell lung cancer)	discontinued clinical development due to low tolerability in phase II studies	[83]
CDK4/6	palbociclib (PD0332991)	human glioblastoma U87 intracranial xenografts and brainstem glioma mouse model	FDA approval for potential treatment of breast cancer	[84,85]
CHK1	UCN-01	A549 (lung carcinoma), NCI-H460 (large-cell lung carcinoma), K562 (erythroblastoid leukemia cell line), glioblastoma stem-like cells *in vitro* and in xenografts	no effect on BEAS-2B (immortalized normal bronchial epithelial cell line) enhanced radiosensitivity of lung cancer cell lines in combination with celecoxib and of head and neck squamous cell carcinoma by combination with ATRA (8 all-trans retinoic acid)	[86,87,88,89]
CHK2	PV1019	MCF-7 (breast carcinoma), U251 (glioblastoma)	radioprotective in mouse thymocytes	[90]
CHK2	XL-844	HT-29 (colon carcinoma)	only one in vitro study with irradiation	[91]
EGFR	cetuximab	several clinical trials combined with standard chemoradiotherapy	FDA approval only for treatment of locally advanced head and neck cancer in combination with radiation	[92,93]
HDAC	LBH589 (panobinostat)	prostate cancer and glioblastoma cells	obatoclax, inhibitor of BCL-2, for increased radiosensitization of glioblastoma cells resistant to LBH589 and SAHA	[94,95,96]
HDAC	PCI-24781 (abexinostat)	cervical and colon carcinoma cells, nasopharyngeal carcinoma cells *in vitro* and in xenografts	two phase I studies as mono- or combination (with doxorubicin) therapy in patients with metastatic carcinoma, lymphomas	[97,98]
				[99,100]
HDAC	SAHA (vorinostat)	LN18 and U251 (glioblastoma cells), osteosarcoma (OS) and rhabdomyosarcoma cell lines and OS xenografts	two finished phase I trials to determine the maximum well-tolerated dose	[101,102,103,104,105,106]
HSP90	17-AAG (geldanamycin)	DU145 (prostate carcinoma), SQ-5 (lung squamous carcinoma), T98G and U87-MG (glioblastoma), esophageal cancer cells	enhanced radiosensitization in combination with the PARP inhibitor olaparib; no radiosensitizing effect in normal tissue cells	[107,108,109]
HSP90	17-DMAG	MiaPaCa (pancreatic carcinoma), NSCLC cell lines	no radiosensitizing effect in normal tissue cells; radioprotective in PBMC	[110,111]
HSP90	NVP-AUY922, NVP-BEP800, NVP-HSP990	various tumor cell lines: A549, GaMG, HT 1080, SNB19, MIA PaCa-2 and U251	no clinical trial	[112,113]
HSP90	STA-9090 (ganetespib)	oropharyngeal squamous cell carcinoma (SCC) tissue samples HCT 116 (colorectal cancer cell line)	effective also in combination with cisplatin and in xenografts combined with capecitabine two ongoing clinical trials in combination with chemoradiation	[114,115]
MDM2	nutlin-3a	prostate cancer cell lines, NSCLC cells	activation of p53 resulted in increased senescence	[116,117,118]
MDM2	PXN727	HCT116 (colon cancer cell line)	upregulation of secretion of HSP70	[118]
MRN-complex	telomelysin (OBP-301)	orthotopic human esophageal cancer xenograft model	ongoing analysis of the safety and efficacy of telomelysin in patients with hepatocellular carcinoma	[119]
p53	PRIMA-1MET MIRA-1	SCLC cell lines with mutant p53 *in vitro* and as xenografts in mouse experiments	reactivation of p53 and radiosensitization	[30]
PRKDC	NU7441	C4-2 and PC3 (prostate carcinoma), MCF-7 SW620 (colon carcinoma) cell culture and xenografts	increased radiosensitization of MCF-7 cells in combination with K55933 no effect in PRKDC-deficient V3 cells	[120,121,122]

Abbreviations: Tergets: ATM (ataxia-telangiectasia mutated), ATR (ataxia-telangiectasia and RAD3-related), BCR-ABL (break-point cluster region-Abelson murine leukemia viral oncogene homolog), CDK (cyclin-dependent kinase), CHK (checkpoint kinase), EGFR (epidermal growth factor receptor), HDAC (histone deacetylases), HSP90 (heat shock protein 90), MDM2 (mouse double minute 2 homolog), MRN (complex of MRE11, RAD50 and NBS1), PRKDC (protein kinase, DNA-activated, catalytic subunit); Substances: SAHA (suberanilohydroxamic acid), 17-AAG (17-*N*-allylamino-17-demethoxygeldanamycin), 17-DMAG (17-Dimethylaminoethylamino-17-demethoxygeldanamycin).

The damage response to ionizing radiation involves activation of the JNK (c-JUN N-terminal kinase) signaling pathway in radiation-sensitive cells [128,129]. The JNK cascade is initiated by MEKK1 (MAP/ERK (mitogen activated protein/extracellular signal-regulated kinase) kinase kinase 1) and requires sequential phosphorylation and activation of MEKK4, JNK and JUN (for a review, see [130]). Activation of the pro-apoptotic JNK pathway may also occur downstream of membrane-derived signals, releasing ceramide [129,131] and DAXX (death associated protein 6), a CD95 binding protein. Binding of HSP27 to DAXX prevents its translocation to the cell membrane and interaction with CD95, resulting again in suppression of apoptosis [132]. Cross-talk exists between the JNK and caspase cascades [133]. In addition to JUN, targets for JNK in the induction of apoptosis include p53, BAX and caspases [134,135].

#### 3.3.3. The Membrane Stress Apoptotic Pathway

In contrast to DNA damage-dependent apoptotic processes, DNA damage-independent apoptotic processes do not require p53. Radiation-induced ROS inflict lipid oxidative damage in the plasma membrane, which results in activation of sphingomyelinase [136,137], followed by rapid hydrolysis of sphingomyelin in the plasma membrane, releasing the second messenger ceramide [138,139,140]. The most important target of ceramide is the RAC1/MEKK pathway, which directly leads to activation of MAPK8 (mitogen-activated protein kinase 8) and of the effector caspases-1, -3 and -6, as well as the autocrine stimulation of the death receptor pathway. MAPK8 has been implicated in apoptosis induced by TNF (tumor necrosis factor) [129]. A second source of ceramide is directly related to DSBs induced by high doses of ionizing radiation, which trigger the activation of ceramide synthase and, thus, the ceramide apoptotic pathway [141,142].

Recent studies suggest that ionizing irradiation can directly induce regulated tumor cell necrosis [143]. Programmed necrosis (necroptosis) displays some overlap with apoptosis. It is a cellular mechanism of necrotic cell death by apoptotic stimuli, *i.e.*, ligand-DR engagement, under conditions where the apoptotic machinery is either deficient or blocked [144].

### 3.4. Mitotic Catastrophe

Mitotic catastrophe has been characterized as the main form of cell death induced by ionizing irradiation [145] and results from premature induction of mitosis before completion of the S and G2 phase [146]. Inhibition or knockout of proteins that control the G2 checkpoint of the cell cycle, like ATM, ATR, CHK1, CHK2 and p21, promote mitotic catastrophe [147,148,149]. It starts with uneven chromatin condensation around the nucleoli, which resembled premature chromosome condensation [150]. Cell death may occur in the first or the subsequent cell division following irradiation. Aberrant mitosis produces an atypical chromosome segregation and cell division causing the formation of giant cells with aberrant nuclear morphology, multiple nuclei or several micronuclei [151,152].

Additionally, radiation-induced mitotic catastrophe is associated with hyper-amplification and overduplication of chromosomes, resulting in multipolar mitosis and subsequent micronuclei formation [152,153,154,155,156]. Centrosome hyper-amplification is a result of DNA damage and compromised DNA repair mechanisms [154]. The centrosome amplification at the G1/S boundary is initiated by the complex of CDK2 and cyclin A or E [157]. Centrosome hyper-amplification is frequently observed in cells lacking functional p53 [158]. In contrast with functional p53, an inhibitor of CDK2, p21, gets activated, inducing cellular senescence (Figure 2).

Mitotic catastrophe can result in cell death either during the M phase, “mitotic death”, or during the following interphase [159]. In some cells, apoptotic pathways are activated during the metaphase, resulting in a delayed type of apoptosis up to six days after irradiation [160,161,162].

Cells that do not undergo mitotic death, but escape mitotic arrest often fail cytokinesis, resulting in a tetraploid DNA content and abnormal nuclei forming giant cells [163]. Giant cells with functional p53 will undergo apoptosis via the BAX-dependent mitochondrial apoptotic pathway during the next G1 phase [164]. However, p53 mutant giant cells can continue several cell cycles and acquire an increasing amount of chromosomal aberrations before they finally die via delayed apoptosis or necrosis [158].

### 3.5. Senescence

Alternative to the induction of apoptosis, activation of p53 by ionizing radiation and subsequent expression of p21 followed by permanent G1-arrest [165,166,167,168,169] may cause senescence [170,171,172]. The senescent cell phenotype is frequently termed stress-induced premature senescence (SIPS) [29,171,173]. In some cases, notably in normal diploid fibroblasts, cells express a prematurely differentiated phenotype that may continue to function over very long time spans [165,174,175,176]. Most p53 wildtype normal human cells, and even many tumor cells, respond to ionizing radiation by undergoing SIPS and not apoptosis [29,167,169,177]. The progeny of irradiated cells accumulate structural chromosomal aberrations in a dose-dependent fashion preceding senescence [178]. Accelerated senescence, like apoptosis, is proposed to be a programmed protective response of the organism to potentially carcinogenic damage [179]. Senescent cells are arrested, but remain viable, metabolically active and are able to secrete factors, including some that may promote tumor growth and progression. However, in the context of tumor cells, it has been proposed that growth arrest may not be irreversible, but induction of senescence might be used by tumor cells in order to escape from radiation-induced cytotoxicity [180]. According to the second view, senescent tumor cells are dormant and might be reawakened by external stimuli, e.g., by factors secreted from tumor stroma cells, months or years after radiotherapy [181].

In normal cells, the p53–p21 and the p16 (CDKN2A)-RB1 pathways act as senescence check points determining terminal growth arrest. The p21-induced G1 cell-cycle arrest is paralleled by p53 suppression of cyclinB1 expression during radiation-induced G2 cell cycle arrest. P16 is also a CDK inhibitor, which acts on CDK4/6, thereby preventing phosphorylation of RB1 (retinoblastoma 1), which remains bound to the transcription factor E2F and, thus, represses the expression of genes required for progression from the G1 to the S phase. Although, the relationship between the two regulatory pathways is still not completely understood, p16-RB1 does not seem to be involved in transient cell-cycle arrest, but may be a redundant backup mechanism for longer-term p21-mediated arrest [182,183] (Figure 2). After induction of the cell cycle arrest in senescent cells, the level of p21 decreases followed by a constitutive upregulation of p16, associating it with the maintenance of growth arrest in senescent cells [184,185]. However, cells without functional p53 will not undergo a permanent cell cycle arrest and senescence, but will die by apoptosis, necrosis, autophagy or mitotic catastrophe [116,186].

### 3.6. Autophagy

Autophagy is a process of self-digestion of organelles in double-membrane vesicles called autophagosomes [187]. In some cells, such lysosomal degradation may be followed by apoptosis, but the surviving cells use the metabolites as energy sources. Autophagy is initiated by a signaling cascade starting with UNC51-like kinase 1 (ULK1). A cytoprotective form of autophagy was described in hypoxic cancer cells leading to digestion of mitochondria damage by radicals. Thus, increased activity of HIF1 (hypoxia-inducible factor 1) further results in increased activity of BNIP3 (BCL2/adenovirus E1B 19-kD protein-interacting protein 3), blocking BCL2, thereby releasing the block of Beclin1, which is an inducer of autophagy [188]. Autophagy plays a dual role during carcinogenesis: development of tumor cells is delayed since damaged mitochondria get eliminated; however, in tumor cells, autophagy is proposed to be responsible for chemo- or radio-resistance by clearing ROS-produced damage [189]. Various studies support the notion that stress-induced damaged organelles or macromolecules are digested before cells enter growth arrest and senescence [190,191].

### 3.7. EGFR (Epidermal Growth Factor Receptor) and PI3K (Phosphatidylinositol 3-Kinase)

Ionizing radiation can mimic the action of ligand binding to EGFR (epidermal growth factor receptor) and was shown to induce receptor dimerization and, subsequently, autophosphorylation at residue T654 [192]. This is followed by receptor internalization [193], compartmentalization into caveolae and translocation into the nucleus [192]. Here, it functions as a transcription factor for the expression of cyclin D1, iNOS (inducible nitric oxide synthase) and B-MYB, facilitating G1/S cell cycle progression and increased proliferation [192,194,195].

**Figure 3:**
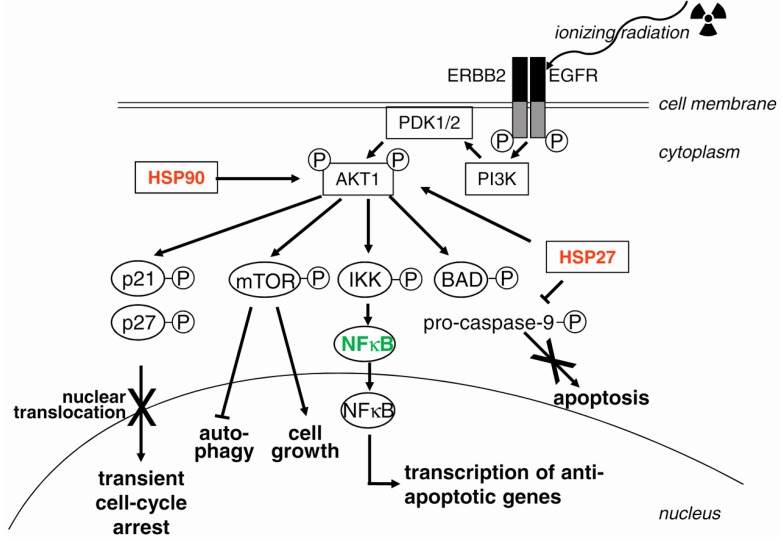
AKT1 as a proliferation and anti-apoptotic factor. Irradiation (symbolized by the sinuous arrow and the trefoil) of heterodimers of EGFR (epidermal growth factor; ERBB1) and of ERBB2 results in activation of PI3K. The subsequently activated PDK1 (pyruvate dehydrogenase kinase 1) and PDK2 phosphorylate and activate AKT1. AKT1 acts as an inhibitor of cell cycle arrest, since it phosphorylates p21 and p27; therefore, both proteins remain in the cytoplasm (indicated by the arrow with a cross) and cannot act as nuclear cell cycle inhibitors. The anti-apoptotic function of AKT1 is fulfilled by the activation of IKK (I-kappa-B kinase), which phosphorylates IκB (I-kappa-B protein, not shown) falling off from the heterodimer with NFκB (nuclear factor kappa-B, subunit 1), which then translocates to the nucleus, inducing transcription of anti-apoptotic genes. Furthermore, Akt1 suppresses apoptosis (indicated by the arrow with a cross) by phosphorylation and inactivation of the two pro-apoptotic proteins BAD (BCL2-associated agonist of cell death) and pro-caspase-9. HSP90 (heat shock protein 90) and HSP27 both stabilize AKT1 and promote degradation of IκB, resulting in enhancement of NFκB activity. HSP27 inhibits cleavage and thus activation of pro-caspase-9. Activation of MTOR (mechanistic target of rapamycin), on the one hand, stimulates cell growth and, on the other hand, inhibits autophagy. Arrows symbolize activation or translocation to the nucleus (described in the text); bar-headed lines symbolize inhibition. Targets for radiosensitization are in red, for radioprotection in green. See text for details.

In response to irradiation, EGFR also functions anti-apoptotically, especially in heterodimers with ERBB2 [196], and transcriptionally activates anti-apoptotic BCL2L1 via SRC and STAT3 (signal transducer and activator of transcription 3) signaling [197]. Furthermore, EGFR–ERBB2 heterodimers lead to activation of the PI3K/AKT1 pathway [198,199] (Figure 3), where AKT1 is activated by PDK1 and PDK2 and, in turn, phosphorylates several target proteins. This results in: (1) activation of IKK (IκB-kinase), which is followed by activation of anti-apoptotic NFκB; (2) inactivation of pro-apoptotic effector proteins, such as BAD and pro-caspase 9 [196]; and (3) suppression of the translocation of the cell cycle inhibitors p21 and p27 to the nucleus. Furthermore, translocated EGFR in the nucleus stimulates the repair of radiation-induced DSB [200,201,202,203].

**Figure 4:**
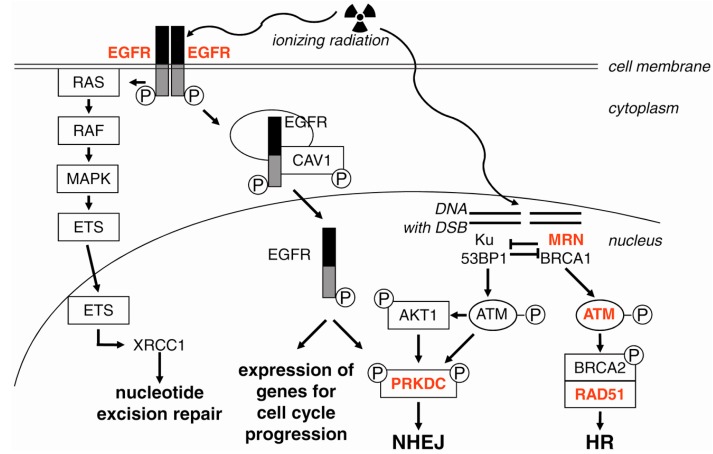
Induction of DNA repair by EGFR signaling. Irradiation (symbolized by the sinuous arrows and the trefoil) results in ligand-independent dimerization of EGFR and autophosphorylation at the cytoplasmic domains, resulting, on the one hand, in activation of the classical RAS/RAF/MAPK pathway, which causes the expression of XRCC1 (X-ray repair cross-complementing group 1) and, thereby, the activation of the nucleotide excision repair. On the other hand, EGFR is internalized in complex with CAV1 and then released into the nucleus, where it functions as a transcription factor for genes required during G1/S progression and activates PRKDC (DNA-PKcs), which plays a pivotal role during NHEJ. AKT1 was also shown as an activator of PRKDC. During the G1 phase, Ku and 53BP1 bind to the DSB (double strand break as symbolized by a gab in the DNA) band activate ATM, which possesses a PI3K domain enabling phosphorylation and activation of AKT1. During the S and G2 phase, the MRN complex and BRCA1 inhibit Ku and 53BP1 and induce DNA repair by homologous recombination (HR). Arrows symbolize activation or translocation to the nucleus (described in the text); bar-headed lines symbolize inhibition. Targets for radiosensitization are in red. See text for details.

Nuclear translocation of EGFR in irradiated cells was accompanied by an increase of Ku70 and Ku80 proteins in the nucleus, indicating activation of non-homologous end joining (NHEJ) [192] (Figure 4). In the nucleus, EGFR physically interacts with and phosphorylates PRKDC, which is required for NHEJ. On the other hand, mutations of the phosphorylation sites of PRKDC caused enhanced cellular sensitivity to ionizing radiation [204]. Phosphorylation of AKT1 by the PI3K domain of ATM resulted in activation of PRKDC and, thus, modulates post-irradiation survival [205,206]. In contrast, inhibition of either AKT1 or upstream regulators of AKT1 prevented radiation-induced phosphorylation of PRKDC [199,207]. In addition, EGFR governs radiosensitivity by affecting the transcription of genes coding for proteins that are involved in base excision repair and nucleotide excision repair. For example, the expression of XRCC1 induced by ionizing radiation is dependent on EGFR/RAS signaling [208,209].

ERBB receptors are also potent stimulators of the RAS/RAF/MAPK pathway. Constant activation of this pathway, *i.e.*, through mutated or constitutively-activated RAS in several tumor types, mediates radiation resistance [210,211]. Mutated RAS proteins not only stimulate the pro-proliferative MAPK pathway, but also the pro-survival PI3K-AKT1 pathway. A constitutively-activated autocrine loop of EGFR-ligand production and receptor stimulation is most likely the reason for the radioresistance of RAS-mutated cells [212,213,214]. Constitutive activity of mutated RAS, especially K-RAS, leads to an enhanced production of EGFR-ligands, e.g., TGFα and amphiregulin. This autocrine EGFR stimulation loop selectively activates EGFR signaling and activation of PRKDC through the PI3K-AKT1 pathway [215]. Activation of mTOR by AKT1 after irradiation mediates the radioresistance of cancer cells. However, resistance to EGFR inhibitors may result from an EGFR-independent aberrant activation of the PI3K-AKT1-mTOR pathway in tumor cells [216]. Furthermore, activation of mTOR antagonizes autophagy [217]. These data indicate that a combination therapy using inhibitors of EGFR and of mTOR might function synergistically for radiosensitization.

## 4. Targets for Radiosensitization

The aim of radiotherapy must be the elimination of all cancer stem cells (CSC) [218]. According to the CSC hypothesis, only these cells are able to proliferate infinitely and repopulate the tumor in contrast to the mass of cancer cells without the potential for self-renewal [219]. Thus, survival of a single CSC will consequently result in recurrence. However, CSC are not only more radioresistant than “normal” tumor cells (non-CSC,) but often acquire a more radioresistant phenotype when exposed to radiotherapy. For example, an increased capability for DNA-repair and for scavenging of ROS was shown for CSC of breast cancer [220], glioblastoma [221,222] and lung cancer [223]. As mentioned above, increased expression and activity of the androgen receptor and hypoxia-induced autophagy result in increased radioresistance of CSC. This obstacle of CSC-inherent radioresistance should be overcome by combining radiotherapy with radiosensitizers.

Many of the proteins acting in the radiation-induced cellular pathways described above are promising targets for either radiosensitizing approaches. Several strategies with different success have been followed to radiosensitize tumor cells and will be described in the following. These include inhibition of DNA (DSB) repair, induction of cell death pathways, suppression of survival pathways or reactivation of p53 (Table 1).

### 4.1. Inhibition of Cell Cycle Control

Interfering with the cell cycle control might result in a loss of G1 or G2/M block, failure of DNA repair and, thus, induction of cell death.

#### 4.1.1. Inhibition of CHK1 or CHK2 (Checkpoint Kinase-1 and -2)

UCN-01 (7-hydroxystaurosporine) was isolated from the culture broth of Streptomyces nearly thirty years ago [224] and has been tested in chemotherapeutic approaches in more than 20 clinical studies (clinicaltrials.gov). In the last 20 years, it has become also a potent radiosensitizer due to its effective inhibition of CHK1 and, thus, abrogation of the G2 checkpoint in different *in vitro* models [86]. Enhanced radiosensitivity was shown in combination with other drugs, like celecoxib or all-trans retinoic acid (ATRA) [87,88,89]. However, only a few xenotransplantations models and no clinical study for the combination with radiation have been published.

Specific inhibition of CHK2 has been achieved by application of the substances PV1019 or XL-844, resulting in significant radiosensitization and reduced proliferation of various cancer cell lines [90,91]. XL-844 promoted mitotic catastrophe [91]. In contrast to its effect in tumor cells, PV1019 had a radioprotective effect in normal mouse thymocytes, abrogating IR-induced apoptosis [90].

#### 4.1.2. Inhibition of Cyclin-Dependent Kinases

Several inhibitors of CDK have already been successfully evaluated in combination with other chemotherapeutics in clinical trials, although negative side effects partially restrict their clinical use drastically [225]. Only a few of these drugs have been tested in combination with irradiation. Inhibition of CDK1 and CDK2 by AZD5438 resulted an increase of IR-induced apoptosis in three NSCLC (non-small cell lung cancer) cell lines by reduced DSB repair by HR [83]. However, the clinical use of AZD5438 for radiosensitization is questionable, since low tolerance to the drug was demonstrated in phase II studies [226].

Downregulation of activated forms of CDK1 and CDK2 was achieved by the application of flavopiridol (alvocidib) before or after irradiation of SEG-1 cells (human esophageal adenocarcinoma), resulting in a G1 arrest and enhanced IR-dependent apoptosis; increased radiosensitivity using flavopiridol was also demonstrated with SEG1 xenografts [82], in a murine glioma model [81] and in radioresistant tumor cells [80]. Due to the disadvantages of flavopiridol, like a narrow therapeutic window, second-generation CDK inhibitors have been developed; dinaciclib already proved its potential in combination with other chemotherapeutic drugs in phase III trials [227], but has not been tested for radiosensitization up to now.

Currently, second-generation pyridopyrimidine-derived inhibitors with high specificity towards CDK4/6 are being tested clinically [228,229]. Palbociclib (PD0332991, PD) has received FDA approval for potential treatment of breast cancer, and abemaciclib (LY2835219) is undergoing a phase III trial for breast and lung cancer. These small-molecule CDK inhibitors cause Rb-dependent G1 arrest in different cancer cells [84,85,230,231]. Few pre-clinical studies have addressed the combination of CDK inhibitors with radiotherapy. Treatment of human glioblastoma U87 intracranial xenografts with PD combined with irradiation (five daily fractions of 2 Gy/fraction) showed increased antitumor activity in growth delay and survival assays [230]. In a brainstem glioma mouse model, seven daily doses of PD after irradiation with a single dose of 10 Gy increased the median survival time [232].

### 4.2. Inhibition of DNA Repair

#### 4.2.1. Targeting the MRN Complex

Telomelysin (OBP-301), an attenuated type-5 adenovirus with oncolytic potency, was used to target the MRN complex as the most upstream sensor in DDR. Infection by this virus resulted in expression of the viral protein E1B55kDa, which in turn led to degradation of the MRN complex and, consequently, in abolishment of ATM activation and DSB repair [119]. A synergistic antitumor effect of three cycles of treatment with telomelysin and regional radiation could be shown in a xenograft model after transplantation of TE8 human esophageal cancer cells [119]. In a clinical phase I/II study, “the safety and efficacy of telomelysin in patients with hepatocellular carcinoma” are currently being analyzed (NCT02293850).

Mirin, another inhibitor of MRN, although with promising *in vitro* results, has not taken the hurdle into the clinic for radiosensitization. *In vitro* experiments already several years ago showed that mirin efficiently blocked direct activation of ATM by MRN, and the exonuclease activity of MRE11, thus, abolished the IR-induced G2/M checkpoint and HR [233].

#### 4.2.2. Targeting ATM (Ataxia-Telangiectasia Mutated) or ATR (Ataxia-Telangiectasia and RAD3-Related)

Mutations in ATM are the cause for the hypersensitivity to ionizing radiation of patients with the autosomal recessive disorder ataxia-telangiectasia. Thus, inhibition of ATM with loss of orderly DSB repair should result in radiosensitization. In two different screens, specific inhibitors of ATM were characterized: KU-55933 [234] and CP466722 [69]. Transient inhibition of ATM by both substances disrupted phosphorylation of the ATM targets CHK2, SMC1 and p53 and furthermore resulted in radiosensitization of HeLa cells; ATR was not affected by these compounds [69,234]. In the last few years, KU-55933 has been tested in various radioresistant tumor cell lines resulting in increased efficient radiosensitization [70,71,72,73]; however, a clinical trial is still pending.

KU-60019, an improved analogue of KU-55933, was 10-fold more effective than its predecessor at blocking ATM phosphorylation [76]. Treatment of glioma or glioblastoma-initiating cells *in vitro* or of orthotopic brain tumors with KU-60019 up to 1 h before irradiation resulted in increased radiosensitization and doubled survival time of the mice, respectively, especially if the glioma cells were mutants for p53 [74,75]. Due to these promising preclinical results and its pharmacodynamics profile, KU-60019 will be probably introduced into clinics in the near future.

ATR is required for the repair of SSB and stabilization of stalled replication forks, which may be converted into DSB. Thus, inhibition of ATR by NU6027 caused *in vitro* radiosensitization of the breast cancer cell line MCF-7 [78]. However, the most promising effects were shown for the combination with various chemotherapeutic drugs. Follow-up studies using also animal models have not been published.

The efficacy of targeting the ATM and ATR pathways is likely to depend on the genetic context. Thus, a high degree of redundancy exists in the mammalian DNA damage response. Synthetic lethality describes the increased sensitivity that arises in cells with two redundant pathways, of which one is non-functional, because of a mutation, and the other pathway is targeted by an inhibitor [235]. One example is the radiosensitization by knock-down or inhibition (KU-60019) of the ATM kinase in a p53-deficient genetic background [75,77].

#### 4.2.3. Inhibition of Homologous Recombination (HR)

RAD51 is an important downstream actor in the regulation of DSB repair by HR. Inhibition of c-ABL by imatinib resulted in decreased expression of RAD51 in various tumor cell lines and, thus, in their reduced clonogenic survival after irradiation with up to 6 Gy [79]. The results of the combination of radiotherapy and imatinib in an animal model with xenotransplantation of PC3 (prostate adenocarcinoma) cells were quite promising: expression of RAD51 was reduced, and the delay of radiotherapy-induced tumor growth was further increased by imatinib; also, very importantly, there was no increased radiation gut toxicity as assayed by determining intestinal crypt cell survival [79].

Inhibition of the function of HSP90 by different substances was also shown to result in increased radiosensitivity of various cancer cells lines. Although the direct target was always HSP90, the radiosensitizing effect was postulated to be induced by different downstream proteins. Use of 17-AAG, (17-*N*-allylamino-17-demethoxygeldanamycin) 24 h before irradiation with doses up to 8 Gy blocked the function of RAD51 and subsequently of HR [107,108]. 17-DMAG (17-Dimethylaminoethylamino-17-demethoxygeldanamycin) reduced the function of the MRN complex and its activation of ATM [110]. Neither substance radiosensitized normal tissue cells [107,108,111]. In fact, 17-DMAG was radioprotective in peripheral blood mononuclear cells by induction of HSP90-dependent stabilization of p53 after irradiation [111]. Three novel HSP90 inhibitors, NVP-AUY922, NVP-BEP800 and NVP-HSP990, probably functioned via interference with cell cycle regulation, resulting either in increased apoptosis [113] or mitotic catastrophe [112] after irradiation. Ganetespib is the only HSP90 inhibitor that is already being tested in two ongoing clinical trials in combination with chemoradiation: either patients with rectal cancer in combination with capecitabine and radiation (NCT01554969) or in patients with esophageal carcinoma in combination with carboplatin, paclitaxel and radiation (NCT02389751).

In recent years, strategies for radiosensitization based on synthetic lethality have been tested clinically. BRCA1 and BRCA2 are involved in the HR repair pathway, but are mutated in familial early-onset breast cancer, rendering this pathway defective. Inhibition of poly(ADP-ribose) polymerase (PARP) sensitizes BRCA-mutated cells, but have less effect on normal cells, which retain a copy of the gene, thus providing the scope for increasing the therapeutic window. PARP is involved in the repair of base damage and SSB by the short-patch BER pathway, and its inhibition is considered to lead to stalled replication forks and DSB formation during replication [236]. However, PARP is also involved in a backup DSB repair pathway, which may be more important [237,238]. Unfortunately, the first PARP inhibitor in a clinical trial did not inhibit PARP at the clinical doses [239]. Nevertheless, a meta-analysis of clinical studies of BRCA-PARP synthetic lethality in combination with various chemotherapeutic agents supports the efficacy of PARP inhibitors in improving progression-free survival, though overall survival was not significantly improved [240]. Fewer studies have combined the synthetic lethal approach with radiotherapy (reviewed in [241,242]), and the outcomes have not been published yet. Synthetic lethality is not limited to BRCA-mutated cancers, since BRCA expression may be repressed by epigenetic factors in sporadic breast cancers [243]. Whether PARP inhibitors will be useful in such cancers or if derepression of BRCA-1 expression and induction of resistance will occur remain to be tested. The principle of synthetic lethality is also being explored for other repair defects than BRCA; thus, an HR inhibitor that shows synthetic lethality in BER-defective tumor cells was recently developed [244].

#### 4.2.4. Inhibition of Non-Homologous End Joining (NHEJ)

Androgen deprivation therapy (ADT) in combination with radiotherapy is currently used for the treatment of prostate cancer patients in the USA with intermediate or high risk tumors [245]. Especially for high risk tumors, an impressive increase in overall survival of up to 78% was demonstrated by ADT for three years starting with the first day of radiotherapy [246]. The combination of ADT with radiotherapy and treatment with docetaxel resulted in men with high risk prostate cancer in an increased overall survival [216].

Targeting PRKDC as an essential component in NHEJ seemed to be a further promising approach for radiosensitization. Several studies have shown the potential of the small molecule NU7441 for significant radiosensitization of various tumor cell lines concomitant with delayed DSB repair and induction of a G2/M block [122,247,248]. No effect could be determined in PRKDC-deficient cells, convincingly demonstrating its specificity for binding to this protein kinase [122]. A combination of NU7441 with the ATM inhibitor K55933 further increased the radiosensitization of MCF-7 cells seen also with both drugs alone [120]. The effect on cell fate seems to be cell line dependent: a combination treatment of irradiation and NU7441 induced in three different NSCLC cell lines three different types of cell death: mitotic catastrophe, autophagy, senescence [248]. In contrast, the combination of NU7441 with K55933 prevented IR-induced senescence of MCF-7 cells and, instead, promoted cell death [120]. This is very important with regard to the long-term negative effects of senescence as mentioned above in 3.5 [249].

#### 4.2.5. Histone Deacetylase (HDAC) Inhibitors

Histone deacetylases (HDACs) are involved not only in deacetylation of chromatin proteins influencing the regulation of gene transcription, but change also the acetylation status of non-histone proteins involved in DSB repair by HR or NHEJ, like p53, ATM, BRCA1, RAD51 and RAD50 [250]. Thus, inhibition of HDAC has been proposed as a promising approach in cancer therapy and for radiosensitization [251]. Indeed, the use of different HDAC inhibitors (HDIs) in several *in vitro* and *in vivo* approaches has supported this hypothesis [251]. In contrast to the first studied HDIs, sodium butyrate and trichostatin A, which exhibited a toxicity profile proscribing their clinical use for some of the successor substances, like SAHA (suberanilohydroxamic acid), LBH589, MS-275, depsipeptide or PCI-24781 (vorinostat, panobinostat, entinostat, romidepsin or abexinostat), showed a much better toxicity profile, enabling their evaluation in clinical studies. However, up to now, only vorinostat has been tested in more than 30 clinical trials for radiosensitization (clinicaltrials.gov). The results of two phase I clinical trials have been published: one with patients with brain metastasis and whole brain radiotherapy and the other with pelvic palliative radiation (PRAVO (Pelvic Radiation and Vorinostat) study) [105,106]. In both studies, the maximum well-tolerated dose of vorinostat has been determined to be a daily dose of 300 mg (five days a week) combined with irradiation with 37.5 Gy in 2.5 Gy fractions over three weeks or with 30 Gy in 3 Gy fractions over two weeks, respectively. A radiosensitizing effect on overall or disease-free survival must be shown in a future phase II trial.

*In vitro* experiments showed that the resistance of different patient-derived glioblastoma cells to SAHA and LBH589 was dependent on a high expression of antiapoptotic BCLXL, which prevents the activation of caspase-3/7. This resistance was overcome by the simultaneous application of obatoclax, an inhibitor of BCL-2 family proteins, resulting in significant net radiosensitization [94]. Analysis according to the effects on normal tissue cells revealed that treatment of human skin fibroblasts or human osteoblasts with SAHA 24 h before irradiation (up to 8 Gy) did not affect their proliferation and survival [104]. Similar results were shown for a normal prostatic epithelial cell line without increased radiosensitivity after treatment with LBH589 compared to two prostate cancer cell lines [96]. Contradictory results were published for the treatment of human skin fibroblasts with SAHA or MS-275 where reduced DSB repair capacity and decreased clonogenic survival were shown in combination with radiotherapy [101].

The recently-published results of the treatment of xenografts of nasopharyngeal carcinomas with abexinostat showed not only enhanced cytotoxic effects of irradiation (1 Gy), but also depletion of RAD51 [97], which suggested the additional use of imatinib to repress RAD51.

### 4.3. Suppression of Survival Pathways

#### 4.3.1. Targeting EGFR

Overexpression of constitutively-active forms of EGFR is common for several cancer types and associated with poor prognosis [209]. The activity of EGFR can be blocked by the use of either antibodies (cetuximab, panitumumab) binding to the extracellular domain and inhibiting receptor dimerization or by small molecules (gefitinib, erlotinib) blocking the intracellular kinase domain. Up to now, most clinical trials to induce increased radiosensitivity have been performed by the application of cetuximab combined with standard chemoradiotherapy [92]. The results have been mostly disappointing, since increased toxicity with cetuximab was not accompanied by improved overall survival compared to standard therapy. Thus cetuximab in combination with radiation has only received FDA approval for the treatment of locally advanced head and neck cancer [93]. Next generation EGFR inhibitors must prove a better toxicity profile and increased radiosensitivity.

Inhibition of the activity of nuclear EGFR is still a challenge, since anti-EGFR therapies did not block the function and translocation of nuclear EGFR. Thus, other proteins that are required for the function of nuclear EGFR must be targeted [252]. *In vitro* results showed that blocking of AKT1 or SRC family kinases resulted in the inhibition of nuclear EGFR translocation and sensitization of cells to anti-EGFR agents [252,253,254]. However, it is still a far way to the clinic.

#### 4.3.2. Induction of Senescence

Inhibition of MDM2 by the small molecules Nutlin-3a or PXN727 caused stabilization of the tumor suppressor p53 and subsequent expression and activation of its target p21 [116,117,118]. This treatment resulted in significant radiosensitization in all tested tumor cell lines with wildtype p53 and reduced proliferation due to induction of the premature senescence of the cancer cells. Induction of senescence might be advantageous for a current cancer treatment; however, accumulation of senescent cells leads to an increased secretion of inflammatory cytokines, which might cause age-related pathologies, like secondary cancers, in the long term [182]. Nevertheless, the primary aim of cancer treatment leading to a longer overall survival should always take preference. Thus, the hypothetical possibility that senescent cells may be dormant with an intrinsic capability to reawaken years after the treatment is of secondary concern, similar to the risk of inducing second cancers.

#### 4.3.3. Inhibition of Autophagy

Prevention of autophagy might result in an accumulation of free radicals, which further should potentiate radiation-induced damage. Chloroquine and hydroxychloroquine have been tested successfully for inhibition of autophagy in various tumor cell types *in vitro* and in animal tumor models. Recently, the results of five clinical trials of hydroxychloroquine combined with different chemotherapeutics were published [255]. The combination with radiotherapy was analyzed only in one study [256]; similarly to a previous report [257], an increase in overall survival was not achieved by additional treatment with hydroxychloroquine, and thus, inhibition of autophagy or increased radiosensitivity could not be proven. The attempts to enhance radiosensitivity by treatment with chloroquine and hydroxychloroquine seem questionable, since it is still not clear if autophagy is indeed induced by radiotherapy in any tumors of patients and functions radioprotectively [180]. Very important with regard to normal tissue effects is the low number of studies addressing radiation-induced autophagy in non-cancerous cells and if delivery of autophagy-inhibiting drugs will be harmful for normal tissue [180]. Further tests are warranted in this field before inhibitors of autophagy will enter the clinic as radiosensitizers.

### 4.4. Reactivation of p53

p53 is mutated in approximately half of all tumors and, thus, is no longer able to function in its pivotal role as a tumor suppressor. In order to identify substances that are able to restore the function of mutant p53, various small-molecule drug screens were performed [30]. Indeed, PRIMA-1 (and its methylated derivative PRIMA-1(MET)) and MIRA-1 are able to restore the correct conformation of specific mutant forms of p53 and, thus, its function as a transcription factor [258,259]. Both substances showed efficient induction of cell death in tumor models and were successfully tested in combination with different chemotherapeutic drugs and in xenotransplantation mouse models [30]. The first clinical trial with PRIMA-1(MET) showed “a favorable pharmacokinetic profile” [260]. However, increased radiosensitivity of p53^Null^ prostate cancer cells after treatment with PRIMA-1(MET) indicated a p53-independent mechanism of its function [261].

## 5. Perspectives

In order to widen the therapeutic window between tumor control and induction of adverse reactions in normal tissue, targeting strategies for increasing CSC radiosensitivity should show differential effects between tumor and normal tissue cells. Drugs targeting cell-cycle progression and checkpoints take advantage of the fact that dose is frequently limited by late-reactions in tissues characterized by resting cells with slow turnover, whereas cancer cells are actively proliferating. Furthermore, tumors are frequently defective in the p53 pathway, whereas normal cells have wildtype p53. Since p53 is important for the intrinsic apoptotic pathway, activating p53 would increase the radiosensitivity of tumor cells without affecting normal cells.

At first sight, targeting DNA repair may not appear a useful approach, since normal cells rely on repair for survival. However, DNA repair pathways form a network, including redundant backup pathways, and the presence of repair defects in certain tumor cells has created opportunities for developing synthetic lethality with small-molecule inhibitors without strong adverse effects in normal tissue. The first example of this strategy was the use of PARP inhibitors, which have shown beneficial effects in clinical trials with chemotherapeutic agents. Trials combining PARP inhibitors with radiotherapy are underway, and new inhibitors and new targets for synthetic lethality are being developed as our understanding of DNA repair mechanisms increases. Because this approach can also be used for targeting cell-cycle checkpoints and because of the inherent difference between tumor and normal cells concerning pathways related to genetic stability, synthetic lethality promises opportunities for increasing the therapeutic window of radiotherapy.

Suppression of survival pathways has so far shown limited clinical success in combination with radiotherapy. The efficacy of targeting the PI3K/AKT pathway directly (e.g., by mTOR inhibitor everolimus) has been tested mostly for the treatment of glioblastoma, but did not show additional survival benefit in a phase II trial [262]. Studies using antibodies against EGFR have had some success in combination with radiotherapy against squamous cell carcinoma of the head and neck [263,264]. There is evidence that redundancy with other receptor tyrosine kinases (RTKs) may prevent efficient inhibition of survival via the PI3K/AKT pathway [265]. Furthermore, the role of EGFR in DNA repair contributes to its radioprotective effect as described above. Therefore, the clinical efficacy of EGFR inhibitors in combination with radiotherapy is likely to depend on the genetic context, and thus, screening of key pathway elements may be crucial for improving outcome. Dual-specificity kinase inhibitors targeting two or more RTKs might prove more efficient than single-specificity inhibitors in enhancing radiosensitivity, but would require active RTKs to be different in tumor cells and normal tissue in order to widen the therapeutic window.

## 6. Conclusions

During the last twenty years, our knowledge of the cellular pathways that are activated in response to ionizing radiation has been drastically increased. However, we are still far from understanding the complete network of interactions and regulatory mechanisms that decide whether a cell will survive or will choose one of the possible cell death pathways. Nevertheless, based on the current knowledge, new therapeutic approaches with highly promising drugs could be developed for radiosensitization of CSC. The opposite approach, radioprotection of normal tissue cells, has been also investigated extensively. This might be achieved by scavenging of ROS, induction of survival, proliferation, self-renewal and differentiation, induction of cell cycle arrest or suppression of apoptosis (for a detailed discussion, see [16,266]). Combining both approaches should create even more opportunities for widening the therapeutic window of radiotherapy as part of a successful multimodality treatment of cancer.
